# In Favour of Medical Dissensus: Why We Should Agree to Disagree About End‐of‐Life Decisions

**DOI:** 10.1111/bioe.12162

**Published:** 2015-04-23

**Authors:** Dominic Wilkinson, Robert Truog, Julian Savulescu

**Keywords:** consensus, withdrawing treatment, intensive care, medical ethics, neuroethics

## Abstract

End‐of‐life decision‐making is controversial. There are different views about when it is appropriate to limit life‐sustaining treatment, and about what palliative options are permissible. One approach to decisions of this nature sees consensus as crucial. Decisions to limit treatment are made only if all or a majority of caregivers agree. We argue, however, that it is a mistake to require professional consensus in end‐of‐life decisions.

In the first part of the article we explore practical, ethical, and legal factors that support agreement. We analyse subjective and objective accounts of moral reasoning: accord is neither necessary nor sufficient for decisions. We propose an alternative norm for decisions – that of ‘professional dissensus’.

In the final part of the article we address the role of agreement in end‐of‐life policy. Such guidelines can ethically be based on dissensus rather than consensus.

Disagreement is not always a bad thing.

## Introduction

A year ago^1^ I was involved in antenatal counselling for a couple, Sean and Susan Cooper. They were in the last trimester of pregnancy, and their foetus had been diagnosed with major congenital abnormalities. Among other problems, it appeared that the foetus had a congenital abnormality of his airway. If he were to survive, he would likely require urgent surgery immediately after birth for insertion of a tracheostomy. Each of the foetus' problems were potentially treatable, though they would require multiple operations. With surgery, the most likely outcome was that the infant would survive. His long‐term functional outcome was difficult to predict, but he would potentially have normal intellectual function. As a counselling neonatologist, my role was to discuss management of the infant after birth. One question was whether or not it was appropriate to offer the option of palliative treatment at birth (with the expectation that the infant would die). I had personally come to the view that surgical treatment would be in the best interests of the infant; I did not feel comfortable withholding life‐sustaining treatment. The obstetric team, however, had reached a different conclusion and felt that palliative care was a reasonable option. What ought I do in this situation? I discussed the case with other neonatal consultants. Most felt as I did that resuscitation (including surgery if necessary) should be provided. One other, whose judgment I respected, supported a palliative approach if that were desired by parents.

When a group of health care professionals are contemplating decisions about end‐of‐life care for incompetent patients do they need to reach *consensus*? One view is that such agreement is a necessary precondition of end‐of‐life decisions.

The ***Professional Consensus Requirement (PCR)**:* Treatment limitation may be discussed with families and may proceed if and only if there is collective agreement within the treating team and endorsement of such a decision.

Is this view correct? Below, we provide evidence that the PCR is included in a number of professional guidelines relating to end‐of‐life decision‐making. Furthermore, consensus forms a key part of accepted practice for development of end‐of‐life policy. Towards the end of the article we will consider the importance of professional agreement in that area. However, consensus may not be a part of end‐of‐life decisions in some parts of the world, or in some areas of medicine. For those who are not favourably inclined towards professional consensus for individual medical decisions, reflecting on the moral significance of agreement or disagreement may shed important light on the problem of physician variability in decisions.

A few points of clarification to start: we are focusing here on end‐of‐life decisions, in particular decisions to discontinue or withhold potentially life‐prolonging treatment. We acknowledge that the same arguments may apply to other ethically contentious decisions, although we will not explore them here. We will also concentrate on incompetent patients (adults or children) without an advance directive. We take it as a given that competent patients may decline treatment without the need for agreement by anyone else. Finally, we are interested in particular in the question of substantive^2^ medical or professional consensus.^3^ We set aside the problem of disagreement between family and medical team, or the difficult practical problem facing clinicians when family members disagree with each other about end‐of‐life decisions.

There are a range of potential approaches to the professional disagreement displayed in the case example. Further discussion between the parties, or further investigations, may clarify the relevant facts and lead to agreement. Ethics consultation might be sought. Indeed, attaining consensus in the face of disagreement is often seen to be a key role of ethics consultation.^4^ We accept that professional agreement is often desirable.^5^ It would be good if agreement were reached about the best course for Sean and Susan Cooper's baby. But is such agreement necessary for treatment limitation to be an ethically permissible option?

## Consensus

Some professional guidelines suggest that in the above example professionals should reach consensus before embarking on any limitations to treatment.

For example, an Australasian Intensive Care Society Statement in 2003 clearly endorsed the Professional Consensus Requirement: ‘Any decision to withdraw or limit treatment first requires the consensus of the intensive care team and the primary medical or surgical team’^6^

In the same year, the UK Intensive Care Society (ICS) guidelines stated:

Ideally, there should be consensus among the entire clinical team who have been heavily involved in the patient's care, that it is appropriate to withhold or withdraw aggressive treatment … Unanimity is desirable but may be unobtainable.^7^

More recently, the General Medical Council has noted: ‘You should aim to reach a consensus about what treatment and care would be of overall benefit to a patient who lacks capacity.’^8^

These guidelines endorse a range of attitudes towards consensus, from it being a mandatory precondition for decisions, through to it being something to aim at. However, before discussing whether consensus is necessary it is worth clarifying what we actually mean by the term.

None of the aforementioned guidelines define ‘consensus’. The word is derived from Latin (consentire, literally: to feel or think together).^9^ Here are two definitions from major dictionaries:

OED: ‘Agreement in opinion; the collective unanimous opinion of a number of persons.’^10^

Merriam Webster: 1. ‘general agreement : unanimity’ or 2. ‘the judgment arrived at by most of those concerned’^11^

These definitions highlight that there are different degrees of agreement. Indeed, fact, we could use ‘consensus’ to refer to at least three different things (see Table 1). At one end of the spectrum lies uniform agreement. The Australian and UK intensive care guidelines appear to use consensus in this way. However, the UK guideline also accepts that unanimity may not be possible. Another Australian guideline, which strongly promotes a ‘consensus building model’, notes that it may be possible to reach decisions with one dissenter:

In circumstances where one team member is in disagreement with the others, the team as a whole should … seek the opinions of professionals from the same discipline as the disagreeing member. In the event that support for this position cannot be found, it may be appropriate for the dissenting member not to continue being involved in the treating team.^12^

**Table 1:** Levels of agreement in decisions

Consensus decision		Proportion favouring ultimate course of action
	1. Unanimous	100%
	2. Near Unanimous	E.g. >90%
	3. Absolute majority	>50%
**Non‐consensus decisions**		
	4. Largest group (non‐majority)	<50%
	5. Minority view	<50%

On this view, consensus might be reached with near‐unanimous agreement.

At the other end of the spectrum, the Merriam Webster definition appears to include majority decisions as also being a type of consensus.

If consensus were necessary for end‐of‐life decisions, it would be important to be clear about the definition, or level of consensus required. However, we will argue that consensus is not necessary on any of these definitions. On our view, even majority support is unnecessary for a decision to be ethically justifiable and a course of action permissible. First things first, though; we should consider why consensus might be important.

### In favour of consensus

One reason to seek consensus in controversial decisions is its practical and psychological value. It is likely to be helpful for families to know that medical professionals concur that life‐sustaining treatment is not in the patient's best interests. Professional agreement (and a clear recommendation) would potentially avoid families feeling as if they are carrying the full burden of decision‐making, and leave them less likely to feel guilty for a decision to discontinue treatment. Conversely, professional disagreement might be distressing for families.^13^ It would potentially make it more likely that families would request that treatment continue.

Consensus may also have value for the clinicians involved. It would potentially help them to feel confident in a decision not to provide treatment.^14^ It might reduce legal vulnerability. Professionals are often concerned about the possibility that an end‐of‐life decision could lead to sanction.^15^ Where all of one's professional colleagues are agreed that it is ethical to withdraw or withhold treatment, it would appear much less likely that the responsible clinicians would be prosecuted. In common law jurisdictions, it would also be less likely that prosecution would be successful. Evidence that other responsible professionals would have taken the same course of action would appear to provide a strong Bolam defence against a negligence claim.^16^

Medical consensus might be thought to be valuable because of its epistemic value.^17^ The 18th century mathematician Nicholas Condorcet pointed out that if decision‐makers individually have at least a 50% of arriving at the ‘correct’ answer, the probability of a collective correct answer will increase (and approach certainty) as group size increases.^18^ The PCR would then improve the chance of a morally correct conclusion. Those more sceptical of moral realism (the concept that moral claims can be true or false), on the other hand, might embrace consensus because it represents ‘the closest we can come to [verifiable truth]… in a context of incomplete knowledge’.^19^

Finally, consensus in end‐of‐life decisions might be of value for society. The ‘sanctity of life’ principle remains strongly endorsed by many societies.^20^ ‘Sanctity of life’ does not mean that life must always be prolonged. However, it has been taken to mean that medical decisions that would lead to earlier death are taken cautiously and carefully. In one paper, describing treatment limitation decisions in newborn intensive care, this was cited as the principle justification of the PCR. ‘If one member of staff felt that treatment should be continued, support was continued. To stop treatment, when it resulted in the death of an infant, was an irrevocable step, whereas if treatment were continued the infant could be reassessed later’.^21^

Some or all of these reasons are likely to be behind the recommendations above. However, there are also several arguments against consensus.

## Against Consensus

The first, and perhaps strongest, argument against the Professional Consensus Requirement is that it imposes the values of physicians upon the patient and the family. End‐of‐life decisions, like all ethical decisions, are based upon both facts and values.^22^ This is one reason why it can be difficult to reach professional consensus: even where there is a common understanding about the relevant facts, professionals may well apply different values. However, if professionals will only limit treatment where there is professional consensus, it means that the only options that will be offered or allowed are those that accord with the shared values of the professionals.

Why is this a problem? There are two distinct tasks involved in end‐of‐life decisions for an incompetent patient. First, there is a need to determine what would be in a patient's best interests, what would best promote their welfare. Second, there is a need to assess what the patient would have wanted, which course would best promote their past autonomy.^23^ Patient values are relevant to the first of these tasks, albeit not determinative. However, patient values (at least for an adult or older child) are essential to the second task. People may have very firm views about how and when they want to die, even if their lives might be worth living.

There is clear evidence from a large number of studies internationally that physicians vary in the values that they apply to end‐of‐life decisions, and, consequently in their decisions.^24^ Decisions are influenced by physician gender, age, religion, and personal views.^25^ Physicians desire less life‐sustaining interventions than patients,^26^ and do not necessarily share religious and cultural values.^27^ Doctors' own preferences for life sustaining treatment appear to influence their perception or assessment of patients' preferences.^28^ In one study, using a simulation of ICU end‐of‐life decision‐making, less than half of the participating physicians treated the patient according to his wishes, despite the simulated patient and his wife having clear preferences about intensive care.^29^ If a unanimous or near‐unanimous consensus threshold is used, the Professional Consensus Requirement would make end‐of‐life decisions hostage to the most conservative decision‐maker(s). One or two professionals with strong views against limitation of treatment might exert a veto over decisions despite the patient and his or her family holding quite different views.

Secondly, there may be more qualms about legal vulnerability where there is disagreement. But a Bolam defence does not require that all, or even a majority of peers would have taken the same course of action. As long as a ‘responsible body of medical opinion’ agrees that such a course was reasonable the action is not negligent.^30^

Finally, although the PCR may set a higher bar for decisions, it is not clear that this is the only way, or the best way to ensure that safe and appropriate decisions are made. Indeed, it seems likely that the PCR would lead to prolongation of potentially futile treatment and delay of inevitable death in at least some patients.

## How Should We Make Moral Decisions?

In order to understand the limits of seeking consensus in ordinary clinical decision‐making it is worth discussing how moral decisions ought to be made.

### Subjectivism

The first type of answer is subjectivist – what a person ought to do is what that person would desire under certain ideal considerations. In his influential book *The Moral Problem*, Michael Smith argues that to determine what he has most reason to do, a person should examine his ultimate desires.^31^

Smith builds on ordinary intuitions about reasons and morality. One such foundational intuition concerns the importance advice has in determining our reasons: ‘If you are unsure what to do in some situation… you should tap into the wisdom of the folk; you should ask advice.’^32^

Smith develops this intuition into a social procedure for determining reasons. Reasons for actions are those actions agreed upon by the rational folk.

A subjectivist rational desire model of moral decision‐making might appear to favour seeking consensus. However, although they may have privileged access to medical facts, other individuals will not necessarily be in full knowledge of personal facts about the patient and his or her values.

Indeed, John Stuart Mill argued that a person's own judgment about which course of action is best for his life is likely to be better than the value judgments of others.^33^ When it comes to patients, they and their families are often better placed to understand social contextual factors, while doctors are better placed to understand biological contextual factors.

The problem for a subjectivist account is that consensus is not necessarily a guide to rationality nor to systematic justification. What matters on the subjectivist account is process, not numbers. Imagine that a majority of professionals support doing A while a minority support doing B. Whether it is reasonable to do either depends on how their judgments are formed, and whether they are systematically justified and informed. A single individual may have a position that is just as justifiable as a whole herd. The mere fact of consensus or a majority position does not imply that such a position is more rational, nor that it better accounts for context sensitivity.

### Objectivism

The second possibility is that there are some things that are good or right, regardless of people's desires for them. This view is central to so‐called ‘objective list’ theories of wellbeing.^34^

If there are true claims about what is objectively good for people, how should we determine which claims are true and which are false? And if we can decide what is good for an individual, how should we decide which course will best promote that end?

One way to answer both these questions is to look at other areas of inquiry. John Rawls described one paradigm of rational reflection in his method of reflective equilibrium.^35^ According to Rawls, the party to rational deliberation should be knowledgeable about the facts and of the consequences of the various courses of action. But they should also be ‘reasonable’. There are four criteria for reasonableness: (i) being willing to use inductive logic, (ii) being disposed to find reasons for and against a solution, (iii) having an open mind, (iv) making a conscientious effort to overcome his intellectual, emotional and moral prejudices.^36^ Lastly, the decision‐maker is to have ‘sympathetic knowledge … of those human interests which, by conflicting in particular cases, give rise to the need to make a moral decision’.^37^

Rawls explicitly rejects the idea that those engaging in rational deliberation should all begin from a similar set of values. Indeed, this is what is at issue: what the values *should be*.

Drawing on Rawls' account, there are two problems for professional consensus in moral decision‐making. First, there is the risk that such a process will bias results because professionals share a common set of values. Medical professionals do not have special insight into what is objectively right. Secondly, professionals will vary in their quality as rational deliberators. Some will be better than others. The fact that a majority exists does not imply that majority is more rational. Indeed, there might be circumstances where there is a less than 50% chance of individual physicians reaching the correct conclusion. If that is the case, Condorcet's theorem implies that larger groups will be *less* likely to yield the right answer.^38^

So on both subjectivist and objectivist accounts of moral decision making, medical consensus is neither necessary nor sufficient for judging what ought to be done.^39^

## Dissensus

Even the least stringent consensus threshold (the absolute majority) would appear vulnerable to the problems we have described. What is the alternative? Table 1 lists two forms of non‐consensus decision‐making. One option would be to use a simple democratic process, permitting end‐of‐life decisions if this receives the highest number of votes (even if an absolute majority is not achieved). However, this is vulnerable to all the problems we have described relating to consensus. Patients who do not share relevant values with the largest group of physicians would still have their options constrained. The final option, furthest from consensus, would be to allow end‐of‐life decisions even if a minority of involved professionals (perhaps only one) believe that such a decision is correct. We could call this model the professional dissensus view:

***Professional Dissensus view***: Treatment limitation may be discussed with families and may proceed where at least one member of the treating team after adequate reflection and discussion, would endorse such a decision (and would be prepared to take over the care of the patient if they are not the responsible clinician).

If we accept this view, professional disagreement would not be an obstacle to discussing limitation of treatment with a family, nor to proceeding to treatment withdrawal if the family feel that this is an appropriate course of action. Indeed, this would be the case even if, as in the example at the start of this article, only a minority support treatment limitation, and even if the treating physician were not one of those who would personally recommend limiting treatment. Given the variability in physician end‐of‐life decision‐making, and the evidence that physician personal values influence such decisions^40^ the physician ought to be prepared to step aside from his or her personal views, and impartially discuss the options with Sean and Susan.

### In favour of dissensus

As already noted, there is a range of different values that patients bring to end‐of life decisions. These values lead individuals to make different decisions. There is epistemic uncertainty, for example, about the effectiveness of treatment, or the patient's quality of life if they survive, or about what exactly the patient would have wanted in a given circumstance. And there is normative (moral) uncertainty, about which moral rules or principles or frameworks should be applied, and in which way.^41^ The important consequence is that we should not seek the single and only right course of action in many cases but rather examine whether there are a range of reasonable courses of action over which patients/families may exercise choice. It is a mistake to think there is always one course, and that a group of clinicians can identify it.

Importantly, embracing dissensus need not assume a simplistic schema of medical‐decision‐making, in which doctors provide the facts while patients supply the values for decisions, and make up their own minds.^42^ The values that patients and family members must draw on may be ‘unexplored and undeveloped…complex, contradictory and confusing’ (ibid). The task of the clinician is not just to *elicit* the patient's (or surrogate's) preferences, but to help patients *construct* preferences that are compatible with their other core commitments and values.^43^ The critical step is for physicians to engage with the patient or their surrogate in rational discussion.

Rational discussion between doctors and patients about which course is best, all things considered, is fundamental to medical decision‐making. Our own conception of what is in our interests is improved by rational discussion with those who share differing conceptions of the good. Each party should present reasons to the other. And in order to determine what constitutes the relevant circumstances of an individual, a rational dialogue is required.^44^ Doctors can yield disease related information; patients or families will describe important psychosocial context.

If the aim of medical decision‐making is knowledge of what is really in the patient's best interests, it is not necessary, nor even advantageous, that all professionals (or patients) begin from a similar set of values. Rational discussion does not require that two parties agree from the outset. Indeed, discussion would be short‐lived if both parties were in complete agreement from the outset.

### Against dissensus

There are a range of potential arguments that might be raised against the dissensus view.

It may be that most families would choose continuation of treatment in the face of divergent professional opinions and that families would find decisions more difficult or burdensome where professionals disagree. Yet, this in itself does not invalidate the dissensus approach. Those patients whose values would be respected by discontinuing treatment have the opportunity to take this option. The solution to difficult decisions is not to take them out of the hands of patients or surrogates.

There might be concerns about whether the Professional Dissensus view would lead to limitation of treatment in situations where this would be inappropriate. For example, some members of the clinical team might endorse limiting treatment at birth in the case example because they are unaware of pertinent facts about the clinical condition. We wouldn't want such factual misunderstandings to count in favour of limiting treatment. However, there are two features of the Professional Dissensus view, as described above, that would work against this possibility. First, we have specified that only views after ‘reflection and discussion’ would be included. Secondly, and importantly, we have suggested that clinicians who endorse treatment limitation be prepared to take over responsibility for the patient's care. This imposes a level of commitment to decisions and would require non‐treating consultants to take the decision just as seriously as the treating consultant. It would also potentially address concerns related to conscientious objection. The treating consultant could potentially ask those members of the team who would support withdrawal or withholding of treatment to take over discussion with the family and management.

We have argued that we should not privilege a professional view, but what safeguards would there be for decisions if professionals disagree? There are several conceivable. Decisions would still need to be consistent with the prevailing law on end‐of‐life decisions. The responsible consultant, on the dissensus view, would remain legally responsible for their decision. Indeed, given that peers take a different view, the physician would potentially hold a greater personal responsibility for action. This would mean that the consultant would need to take additional care to justify their decision and to clearly document their reasons for doing so.^45^ Further, we could build in additional processes for decisions made in the absence of agreement. For example, ethics consultation might be mandatory in such situations. However, the aim of such consultation would not be to achieve collective agreement – rather to ensure that the decision is consistent with prevailing policy or law, that all appropriate factors have been considered, and that the proposed limitation of treatment is a reasonable course of action.^46^

There might be a concern that the Professional Dissensus view does not solve the problem of a mismatch between professional and patient values. For example, none of the clinical team may have values that are aligned with those of the patient and family.^47^ It wouldn't provide a solution in non‐team situations, where only a very small number of medical professionals are directly responsible for the care of the patient. We accept this concern. A separate solution would be required for those situations.^48^ Part of the answer may lie in the development of guidelines for end‐of‐life decisions that draw on a broader range of values (see below). In any case, the dissensus view would still provide a better solution to the problem of mismatching values than the consensus view.

Finally, some might be concerned that the Professional Dissensus view does not solve the problem of professional disagreement; it merely shifts the goalposts, or changes the focus of debate. In the case example, disagreement between neonatologists and obstetricians might shift from a question of whether palliative care would be in the best interests of the infant, to whether palliative care at birth would be legal, or would be a ‘reasonable’ option. This is an important point. There will still need to be some limits to decision‐making (for example on the basis of limited resources). There will still be some difficult cases where it is unclear whether or not treatment limitation is an available option. However, we suggest that recognition of the variability of physician views about treatment, as well as the importance of patient values will help make decisions possible despite disagreement. We will turn next to the role of professional consensus (or dissensus) in setting out the limits to ‘reasonable’ decisions.

## Dissensus and End‐of‐Life Policy/Guidelines

We have alluded to two potential roles for end‐of‐life policy or guidelines. Such guidelines may be useful in situations where there is no professional disagreement, but where the values of the patient differ from those of available professionals. In such circumstances, setting out which options ought to be available may help avoid misplaced paternalism. Guidelines are also necessary to set out the boundaries of permissible decision‐making, to work out when particular professional views should not be respected. What role should consensus play in policies relating to provision of life‐support, how much agreement is necessary?

### Policy example: Resuscitation of extremely premature infants

When delivery of an extremely premature infant is anticipated neonatal doctors meet with parents to discuss how the infant will be managed at birth. There are multiple published international guidelines to assist doctors in counselling. Many provide indications of the circumstances in which it would be mandatory or optional or unreasonable to provide active resuscitation at birth.^49^ For example guidelines often indicate that resuscitation should not be provided for infants born before 23 weeks of gestation.

Guidelines relating to the care of extremely premature infants have often been established by professional consensus. For example in one region of Australia a guideline was published in 2006 based on a consensus workshop.^50^ Recommendations were seen to have ‘unequivocally reached’ consensus if more than 90% of participants agreed with them and incorporated into guidance if at least 75% agreed with them. They recommended that resuscitation be guided by discussion with parents for premature infants born between 23 and 25 weeks gestation.

However, when the American Academy of Paediatrics reviewed their previously published guideline relating to resuscitation of extremely premature infants in 2009 they did not give specific guidance. Previous statements relating decisions to specific gestational ages were removed. They were only able to provide this vague statement:

When the physicians' judgment is that a good outcome is reasonably likely clinicians should initiate resuscitation.^51^

In a separate commentary, the chair of the committee reflected on the difficult process of achieving consensus. ‘[D]espite long discussions it became apparent that [committee] members could not agree on the precise morbidity and mortality thresholds for [resuscitation]’^52^

Similar guidelines have been developed for policy on admission to intensive care, withholding of life‐sustaining treatment, provision of cardio‐pulmonary resuscitation, etc.^53^ These guidelines appear to have drawn on a version of the Professional Consensus Requirement – they have sought (to a variable degree) a high level of agreement in the development of policy. Yet, there are at least two challenges to a Professional Consensus Requirement for end‐of‐life policy. As would be clear from the discussion of individual decisions, professional consensus necessarily draws upon the values of professionals. These may be shared by broader society, but may also be quite different from those of individual patients. It is unclear that it is justified to set these professional values as determinative given the plurality of values. Second, as the American Academy example illustrates, the perceived need to achieve consensus on such decisions can mean that it becomes impossible to develop specific guidelines. There is an intrinsic tension in guideline writing between vagueness and precision.^54^ The problem of difficulty in reaching consensus tends to encourage vague guidelines that can be endorsed by all, but which are practically unhelpful.

We argued above in favour of the Professional Dissensus view for individual end‐of‐life decisions. Is there an analogous solution for end‐of‐life policy? Here is a parallel proposal:

***Professional Dissensus approach to policy***: Treatment limitation (or provision) should be considered and may proceed where at least some members of the professional community would endorse such a decision after reflection and discussion and would be prepared to provide this care if they were the responsible clinician.

The advantage of this approach over a consensus requirement is that it accepts rather than ignores the existing variation in values and practice relating to end‐of‐life decisions.^55^ Given this variation, decisions should be based on the values of the patient rather than those of the physician who happens to have been rostered to look after them, or the shared values of a group of physicians. For extremely premature infants, this view would mean that policies describe and endorse a range of practice rather than a consensus view. It would be reasonable to offer resuscitation or non‐resuscitation to an extremely premature infant of 23 to 24 weeks gestation if at least some professionals would endorse this.^56^ The boundaries of treatment lie where no professionals would reasonably be prepared to offer treatment, or where all would consider treatment mandatory.

Are there any potential concerns about a dissensus approach to policy? Some might be similar to concerns raised for the dissensus view in individual decisions. A group of professionals may have a distinctive shared set of values, or fail to include representatives of minority groups within a community who have different relevant values. In that case, there will remain a risk of physicians unreasonably imposing their values on patients. Again, a dissensus approach to policy would still be better than the consensus requirement, but additional steps may be needed. For example, any local guideline development should take into account the range of options provided by other institutions, or regions or countries, as well as the range of values expressed by patients in their community. Guidelines should incorporate input from other groups, including, for example, patient groups, ethicists.

Some members of the professional community may have idiosyncratic views, and be prepared to limit treatment in a much wider range of cases than their peers. While the PCR leaves decisions dependent on the most conservative physician, the Dissensus approach might be thought to be hostage to the most liberal views within the medical community. However, this concern may be overstated. The views expressed by professionals must still be consistent with prevailing law and considerations of distributive justice, and must reflect decisions that they would personally be prepared to make. If those decisions are based on sound reasons that the clinician is able to articulate and defend, it appears appropriate for policy to allow this. In any case, treatment limitation would only proceed where the patient (or her parents in a paediatric setting) shared those values, and this would potentially be very uncommon if the views of the physician are idiosyncratic.

One concern might be that the dissensus approach could be seen to encourage variation in practice, and be contrary to the important policy goal of articulating what is taken to be best practice. However, the dissensus approach to policy may be used to generate substantive (rather than procedural) and precise (rather than vague) guidance. In the example of premature infants, evidence that some clinicians offer the option of resuscitation (or non‐resuscitation) at these specific gestational ages could be used to specify the professional standard (ie that between these gestational ages it would be appropriate to discuss the option of resuscitation or non‐resuscitation with parents). Moreover, it is consistent with the dissensus approach to promote or encourage one particular alternative (where there is good evidence or argument to do so). Yet, where there is professional disagreement about how to respond to this evidence or these arguments, policy should reflect this.

A more significant problem for the dissensus approach to policy is that there may need to be limits to the values that can be endorsed. Whether any view can be excluded *tout court* will involve evaluation of its genesis and whether it conforms to standards of rationality. More practically, resource limitations must necessarily override the views of individual physicians. For example, some physicians may be prepared to provide resuscitation and continued intensive care even in the face of extremely poor prognosis and no prospect of benefit. However, public health systems cannot give patients (or physicians) free and unrestricted access to expensive medical treatments. Some values cannot be respected. The classic ‘Harm principle’ justifies limits to individual liberty on the grounds of harm to others.^57^ Providing intensive medical treatments with low or no possibility of benefit harms other patients who are thereby deprived of access to treatment. The important question, then, is about how we decide on resource limits for life‐sustaining medical treatment. Whose values will such limits reflect, and what level of agreement should we seek in deriving them? An adequate answer to that question would be the subject for another article. Nevertheless, it is clear that the values of the patient cannot tell us where resource‐based lines should be drawn. It is also clear that while the views of professionals may be relevant, our answer to questions about resource allocation should also reflect the range of views and values of wider society.

## Conclusions

In this article we have argued that a desire to seek medical consensus is understandable but at present, given our existing moral knowledge, problematic both at an individual patient and at a policy level. It is not supported either by subjectivism or objectivism about reasons. It is not supported by more patient‐centred, non‐paternalistic models of the doctor patient relationship, such as liberal rationalism and sophisticated forms of shared decision‐making. Value pluralism and moral uncertainty in end‐of‐life decisions mean that it is unrealistic and counterproductive to seek unanimous or even majority level agreement. We should more willingly embrace moral uncertainty and difference, and allow a diversity of practice. These experiments in living (or dying) should be appropriately centred around the patient's own values, and on what is good for specific individual patients, not necessarily on what a narrow group of professionals divine is right.

We have argued against the Professional Consensus Requirement for end‐of‐life decisions understood as a decision that the majority would endorse. Nevertheless, agreement may be valuable for a number of reasons. It may still be appropriate to aim for consensus where possible. The process of reasoned discussion, elucidation of facts, and exploration of values is worthwhile even if agreement is not forthcoming. One way to reach consensus in a broader range of cases would be to reframe it as a shared understanding of the range of reasonable decisions that may be acceptable. On this account professionals should agree to respect views that they do not personally share. Professional guidelines should set out the range of views and options that may be reasonably respected, at least in part to address the problem of mismatch between patient and professional values.^58^

In cases like the one described at the start of this article, physicians should be encouraged to engage in rational discussion about values both with their peers and with their patients. One way of dealing with the different views of members of the team would be joint counselling. Those in favour and against the option of palliative care could (and arguably should) sit down together with Sean and Susan, and present the available options and the reasons for their different conclusions. This would potentially help Sean and Susan to understand the nature of disagreement, to fairly appraise their options, and to identify and articulate their own views.

End‐of‐life decisions are, by their nature, difficult, unsettling and sometimes distressing. Professionals, understandably, have different views about them, and will sometimes reach different conclusions. However, such disagreement is not necessarily a sign that we are on the wrong track, and should not be taken to preclude withholding or withdrawing treatment if that is consistent with the patient's/family's wishes.

Let's agree to disagree.

## Footnotes

1
This case is a composite of several real cases encountered by DW. All identifying details have been omitted or changed.

2
Some authors distinguish between substantive and procedural consensus (ie between the content of decisions and the process of decision‐making). BayertzK., editor. The concept of moral consensus : the case of technological interventions in human reproduction. Dordrecht; Boston; London: Kluwer Academic Publishers; 1994. We will focus on (and criticize) the notion of substantive consensus, while ultimately endorsing procedural consensus. (We are grateful to an anonymous reviewer for this point).

3
We will use the term ‘medical staff’ to include both doctors and nurses, as well as possibly other key members of the clinical team caring for a child K.M. Melia . Ethical issues and the importance of consensus for the intensive care team. Soc Sci Med 2001; 53: 707–719.1151104710.1016/s0277-9536(00)00381-6

4

J.D. Moreno . Ethics by committee: the moral authority of consensus. J Med Philos 1988; 13: 411–432; 324658010.1093/jmp/13.4.411 S.A. McLean . What and who are clinical ethics committees for? J Med Ethics 2007; 33: 497–500.1776181510.1136/jme.2007.021394PMC2080817

5

T. Koch & M. Rowell . The dream of consensus: finding common ground in a bioethical context. Theor Med Bioeth 1999; 20: 261–273.1047431210.1023/a:1009995919835

6

Australian and New Zealand Intensive Care Society . 2003 Statement on withholding and withdrawing treatment Version 1. Melbourne. Available at: http://www.anzics.com.au/downloads/doc_download/136‐the‐anzics‐statement‐on‐withholding‐and‐withdrawing‐treatment‐version‐1 [Accessed 21 June 2013].

7

Intensive Care Society . 2003 Limitation of treatment on ICU. London. Available at: http://www.ics.ac.uk/EasysiteWeb/getresource.axd?AssetID=469&type=full&servicetype=Attachment [Accessed 21 June 2013].

8

General Medical Council . 2010 Treatment and care towards the end‐of‐life: good practice in decision making. London: GMC.

9

Oxford English Dictionary . 2013 ‘consensus, n.’: Oxford University Press.

10
ibid.

11

Anonymous . 2014 Merriam‐Webster. Available at: http://www.merriam‐webster.com/dictionary/consensus [Accessed 29 Jan 2014].

12

NSW Department of Health . 2005 Guidelines for end‐of‐life care and decision‐making. Sydney, NSW, Australia. Available at: http://www0.health.nsw.gov.au/policies/gl/2005/GL2005_057.html [Accessed 19 Jul 2011].

13

K.L. Meert , S.E. Eggly , M. Pollack , K.J.S. Anand , J. Zimmerman & J. Carcillo , et al. Parents' perspectives on physician‐parent communication near the time of a child's death in the pediatric intensive care unit. Pediatr Crit Care Med 2008; 9: 2–7; 1847790610.1097/01.PCC.0000298644.13882.88PMC3198033 E.C. Meyer , M.D. Ritholz , J.P. Burns & R.D. Truog . Improving the quality of end‐of‐life care in the pediatric intensive care unit: parents' priorities and recommendations. Pediatrics 2006; 117: 649–657.1651064310.1542/peds.2005-0144

14

Melia , op. cit. 3

15

A. Meisel , L. Snyder & T.E. Quill . Seven legal barriers to end‐of‐life care: myths, realities, and grains of truth. JAMA 2000; 284: 2495–2501.1107478010.1001/jama.284.19.2495

16
In a famous case in UK medical law, (referred to colloquially as ‘Bolam’), a judge ruled that doctors would not be found guilty of medical negligence if they had acted in a way that was ‘accepted by a responsible body of medical men’ K. Mason & G. Laurie . Mason and McCall Smith's Law and Medical Ethics. 8th edn Oxford: Oxford University Press; 2013 p. 148.

17

R. Tong . The Epistemology and Ethics of Consensus – Uses and Misuses of Ethical Expertise. J Med Philos 1991; 16: 409–426.189502510.1093/jmp/16.4.409

18

T. Koch & M. Ridgley . The Condorcet's Jury Theorem in a bioethical context: The dynamics of group decision making. Group decision and negotiation 2000; 9: 379–392.

19
Ibid: 382.

20

D.J. Baker & J. Horder . The sanctity of life and the criminal law : the legacy of Glanville Williams. Cambridge: Cambridge University Press; 2013.

21

A. Whitelaw . Death as an option in neonatal intensive care. Lancet 1986; 2: 328–331.287433610.1016/s0140-6736(86)90014-0

22

R.A. Hope , J. Savulescu & J. Hendrick . Medical ethics and law : the core curriculum. 2nd edn Edinburgh: Churchill Livingstone Elsevier; 2008; J. Savulescu . Rational non‐interventional paternalism: why doctors ought to make judgments of what is best for their patients. J Med Ethics 1995; 21: 327–331; 877845510.1136/jme.21.6.327PMC1376828 J. Savulescu . Liberal rationalism and medical decision‐making. Bioethics 1997; 11: 115–129.1165479110.1111/1467-8519.00049

23
This second task is not relevant to patients who have never been competent, such as infants.

24

D.J. Wilkinson & R.D. Truog . The luck of the draw: physician‐related variability in end‐of‐life decision‐making in intensive care. Intensive Care Med 2013; 39: 1128–1132; 2343595110.1007/s00134-013-2871-6 D.W. Frost , D.J. Cook , D.K. Heyland & R.A. Fowler . Patient and healthcare professional factors influencing end‐of‐life decision‐making during critical illness: a systematic review. Crit Care Med 2011; 39: 1174–1189.2133612710.1097/CCM.0b013e31820eacf2

25

R. Löfmark , T. Nilstun , C.M. Cartwright , S. Fischer , A. van der Heide & F. Mortier . Physicians' experiences with end‐of‐life decision‐making: survey in 6 European countries and Australia. BMC Medicine 2008; 6: 4.1826973510.1186/1741-7015-6-4PMC2277432

26

G.P. Gramelspacher , X.‐H. Hua Zhou , M.P. Hanna & W.M. Tierney . Preferences of physicians and their patients for end‐of‐life care. J Gen Intern Med 1997; 12: 346–351; 919225110.1046/j.1525-1497.1997.00058.xPMC1497117 C.L. Sprung , S. Carmel , P. Sjokvist , M. Baras , S.L. Cohen & P. Maia , et al. Attitudes of European physicians, nurses, patients, and families regarding end‐of‐life decisions: the ETHICATT study. Intensive Care Med 2007; 33: 104–110; 1706628410.1007/s00134-006-0405-1 J. Gipson , G. Kahane & J. Savulescu . Attitudes of Lay People to Withdrawal of Treatment in Brain Damaged Patients. Neuroethics 2014; 7: 1–9.2460048510.1007/s12152-012-9174-4PMC3933752

27

C.L. Sprung , P. Maia , H.‐H. Bulow , B. Ricou , A. Armaganidis & M. Baras , et al. The importance of religious affiliation and culture on end‐of‐life decisions in European intensive care units. Intensive Care Med 2007; 33: 1732–1739.1754155010.1007/s00134-007-0693-0

28

L.J. Schneiderman , R.M. Kaplan , E. Rosenberg & H Teetzel . Do physicians' own preferences for life‐sustaining treatment influence their perceptions of patients' preferences? A second look. Camb Q Healthc Ethics 1997; 6: 131–137.917940710.1017/s0963180100007751

29

A.E. Barnato , H.E. Hsu , C.L. Bryce , J.R. Lave , L.L. Emlet , D.C. Angus & R.M. Arnold . Using simulation to isolate physician variation in intensive care unit admission decision making for critically ill elders with end‐stage cancer: a pilot feasibility study. Crit Care Med 2008; 36: 3156–3163.1893669410.1097/CCM.0b013e31818f40d2PMC2853191

30
See 16 Even if there is professional consensus, the court may judge that a particular action was not reasonable Mason et al. p. 149–150.

31

M. Smith . The moral problem. Oxford: Blackwell; 1994.

32
Ibid: 151.

33

J.S. Mill . On Liberty. New York: P.F. Collier & Son; 1909. Chapter 4

34

J. Griffin . Well‐being : its meaning, measurement and moral importance. Oxford: Clarendon; 1986 p. 40–72; D. Parfit . Reasons and persons. Oxford: Oxford University Press; 1984 p. 499; G.E. Moore & T. Baldwin . Principia ethica. Rev. edn. Cambridge: Cambridge University Press; 1993. Chapter VI. Objective list theorists include, among others, Aristotle, Plato, Aquinas, Leibniz, Adam Smith, Hegel, Marx and Nietzsche.

35

J. Rawls . A theory of justice. Rev. edn. Oxford: Oxford University Press; 1999.

36

J. Rawls . Outline of a decision procedure for ethics. Philosophical Review 1951; 60: 177–197, 179.

37
Ibid.

38

Koch & Ridgley , *op. cit* 18. The phenomenon of ‘groupthink’, may also lead astray consensus decisions. L.L. Janis . Groupthink: psychological studies of policy decisions and fiascoes. 2nd edn Boston: Houghton Mifflin; 1982.

39
The significance of subjective and objective reasons is further discussed in J. Savulescu . Desire‐based and value‐based normative reasons. Bioethics 1999; 13: 405–413.1165812910.1111/1467-8519.00168

40

Wilkinson & Truog , *op. cit.* 24.

41

T. Lockhart . Moral uncertainty and its consequences. New York; Oxford: Oxford University Press; 2000.

42

C.E. Schneider . The practice of autonomy : patients, doctors, and medical decisions. New York; Oxford: Oxford University Press; 1998 p. 69

43

R.M. Epstein & E. Peters . Beyond information: exploring patients' preferences. JAMA 2009; 302: 195–197.1958435110.1001/jama.2009.984

44
‘Liberal Rationalism’ Savulescu 1997, *op. cit.* 22. 10.1111/1467-8519.0004911654791 See also Savulescu 1995, *op. cit.* 22.

45
On this basis, a decision reached in the absence of consensus might be safer than one reached with mutual agreement. As already noted, collective decisions can sometimes give an illusion of security.

46
Ethics committees vary in their legal expertise, and in some institutions legal questions are dealt with by a separate process McLean , *op. cit.* 4.

47
This might occur, for example, with a non‐religious patient being cared for in a religious hospital. K.A. White . Crisis of conscience: reconciling religious health care providers' beliefs and patients' rights. Stanford Law Rev 1999; 51: 1703–1749.10558539

48
For example, it might require solo physicians to audit their end‐of‐life decisions, and to be made aware of their practice in comparison with others Wilkinson & Truog , *op. cit.* 24. It might mean that professionals facing possible end‐of‐life decisions (in situations where they would not personally endorse limitation of treatment) deliberately seek second opinions, or ethics consultation, in order to ensure that the physician's values are not imposed on the patient.

49

M.S. Pignotti & G. Donzelli . Perinatal care at the threshold of viability: an international comparison of practical guidelines for the treatment of extremely preterm births. Pediatrics 2008; 121: e193–e198; 1816653810.1542/peds.2007-0513 D.J. Wilkinson . Gestational ageism. Arch Pediatr Adolesc Med 2012; 166: 567–572.2266503110.1001/archpediatrics.2011.1262

50

K. Lui , B. Bajuk , K. Foster , A. Gaston , A. Kent & J. Sinn , et al. Perinatal care at the borderlines of viability: a consensus statement based on a NSW and ACT consensus workshop. Med J Aust 2006; 185: 495–500.1713745410.5694/j.1326-5377.2006.tb00664.x

51

D.G. Batton & C.o.F.a. Newborn . Clinical report–Antenatal counseling regarding resuscitation at an extremely low gestational age. Pediatrics 2009; 124: 422–427.1956432910.1542/peds.2009-1060

52

D. Batton . Resuscitation of extremely low gestational age infants: an advisory committee's dilemmas. Acta Paediatr 2010; 99: 810–811.2021903310.1111/j.1651-2227.2010.01753.x

53
Consensus report on the ethics of foregoing life‐sustaining treatments in the critically ill. Task Force on Ethics of the Society of Critical Care Medicine. Crit Care Med 1990; 18: 1435–1439; Consensus statement on the triage of critically ill patients. 224562210.1097/00003246-199012000-00026 Society of Critical Care Medicine Ethics Committee. JAMA 1994; 271: 1200–1203; 7818629 R.D. Truog , M.L. Campbell , J.R. Curtis , C.E. Haas , J.M. Luce & G.D. Rubenfeld , et al. Recommendations for end‐of‐life care in the intensive care unit: a consensus statement by the American College of Critical Care Medicine. Crit Care Med 2008; 36: 953–963; 1843128510.1097/CCM.0B013E3181659096 M.F. Hazinski , J.P. Nolan , J.E. Billi , B.W. Böttiger , L. Bossaert , A.R. de Caen , et al. Part 1: Executive summary: 2010 International Consensus on Cardiopulmonary Resuscitation and Emergency Cardiovascular Care Science With Treatment Recommendations. Circulation 2010; 122: S250–S275.2095624910.1161/CIRCULATIONAHA.110.970897

54

J. McMillan , T. Hope & D. Wilkinson . Precision and the rules of prioritization. Camb Q Healthc Ethics 2013; 22: 336–345.2391625010.1017/S0963180113000182

55

Wilkinson & Truog , *op. cit.* 24.

56

Wilkinson op cit. note *op. cit.* 49.

57

Mill , *op. cit.* 33.

58

R.M. Veatch . Abandoning informed consent. Hastings Cent Rep 1995; 25: 5–12.7782200

